# Supplementary material for: Non-invasive ultrasonic neuromodulation of the human nucleus accumbens impacts reward sensitivity
Source: Nat Commun. 2025 Nov 27;16:10192. doi: 10.1038/s41467-025-65080-9 (PMC12660320; doi:10.1038/s41467-025-65080-9)
Supplement: Supplementary file 1 — Supplementary Information [file 41467_2025_65080_MOESM1_ESM.pdf]

Supplementary Material

Non-invasive Ultrasonic Neuromodulation of the Human Nucleus Accumbens Impacts Reward Sensitivity

Siti N. Yaakub[1,2]*, John Eraifej[3-5]*, Nadège Bault[1,2]*, Mathilde Lojkiewiez[1,2], Elouan Bellec[1,2], Jamie Roberts[6], Noah S. Philip[7,8], Amir Puyan Divanbeighi Zand[4], Alexander L Green[3,4], Matthew F.S. Rushworth[9,10+], Elsa F. Fouragnan[1,2+]

* Equally contributing + Joint supervision

Correspondence and material requests should be addressed to Elsa F. Fouragnan (elsa.fouragnan@plymouth.ac.uk).

Supplementary Results

TUS-dACC TUS and decision-making under low reward expectancy

The task design included trials where participants chose between two low-probability (30%) options, creating conditions of low expected value. These trials provide an opportunity to assess whether dACC stimulation modulates behaviour in contexts where adaptive choice requires evaluating similarly unrewarding alternatives. Previous studies in both humans[1,2] and macaques[3] suggest that the dACC is engaged in representing the value of counterfactual alternatives - options not chosen but potentially informative for future switching. Notably, in macaques, TUS-induced perturbation of dACC function abolishes the typical relationship between counterfactual value and behavioural change[3].

Building on this, we explored whether TUS-dACC might impair the brain's ability to recognize that the currently unchosen option is not meaningfully better than the chosen one. As a result, individuals might rely more heavily on broader indicators of low environmental reward—potentially mediated by dorsal raphe mechanisms [4,5] — and increase switching behaviour even when no advantageous alternative is available.

To test this, we conducted a targeted analysis of trials involving two low-probability stimuli. The results revealed that, after TUS-dACC, participants were more likely to switch choices following unrewarded outcomes in these trials compared to Sham. This finding supports the hypothesis that dACC stimulation disrupts the specific valuation of immediate alternatives, leading to greater reliance on global motivational signals that favour behavioural exploration. It further illustrates the nuanced role of dACC in value-guided learning and decision-making, particularly under conditions of ambiguity or uniformly low reward.

Supplementary Figure 1

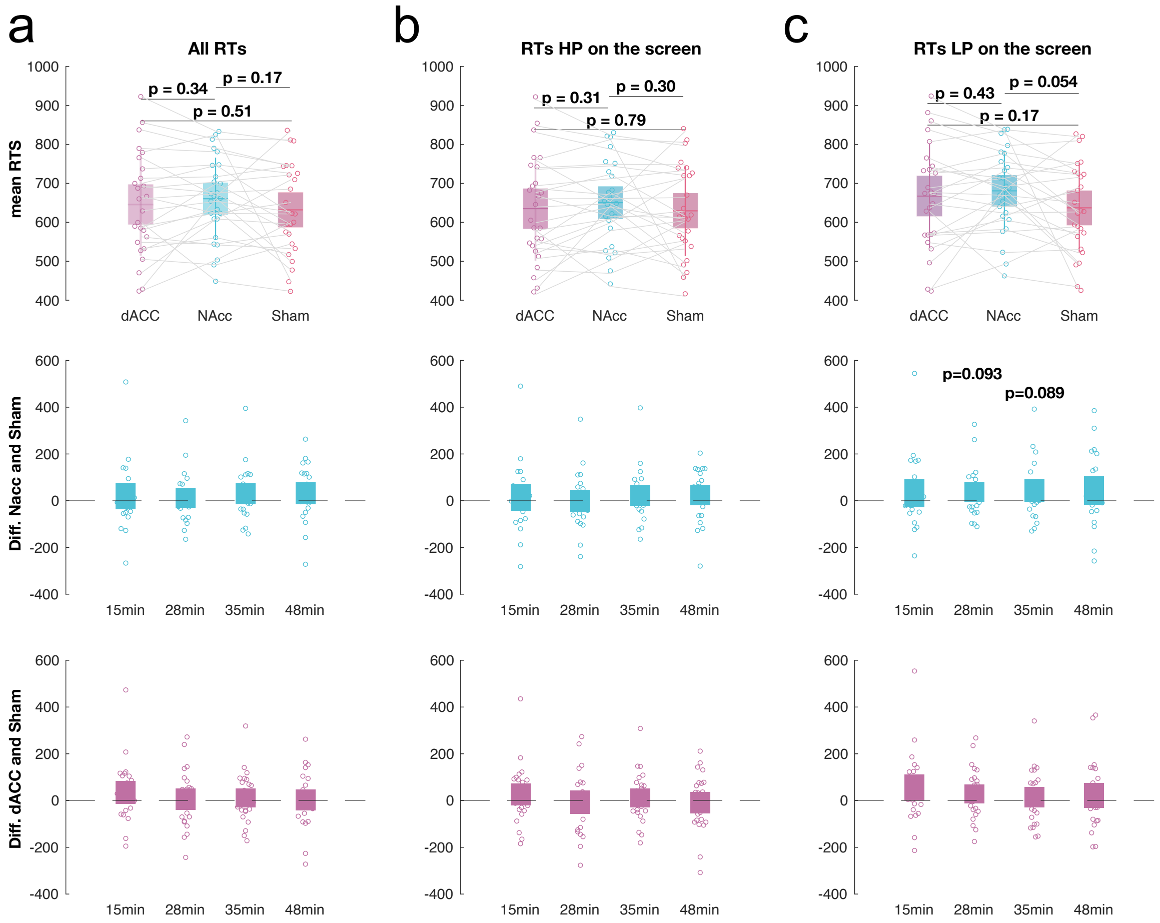

Suppl. Fig. 1. Reaction time analyses across stimulation conditions and trial types. Top row: Mean reaction times following TUS-dACC, TUS-NAcc, and Sham for all trials (left), HP trials (middle; trials with one high-probability and one low-probability option), and LP trials (right; trials with two low-probability options). Each dot represents an individual participant's mean across the task for the specified condition (n=26). Boxes represent the standard deviation around the mean. **Middle row:** Reaction times across the four post-TUS blocks (~15, 28, 35, and 48 minutes) as a difference between NAcc and Sham. Each dot represents an individual participant's mean (n=26). Boxes represent the standard deviation around the mean. **Bottom row:** Reaction times across the four post-TUS blocks (~15, 28, 35, and 48 minutes) and as a difference between dACC and Sham. Each dot represents an individual participant's mean (n=26). Boxes represent the standard deviation around the mean. For **a,b,c top row** statistical significance was determined using One-way ANOVA and two-sided t test. For **a,b,c middle and bottom row**, single two-sided t-tests were employed for each window. No multiple comparisons were applied.

Supplementary Figure 2

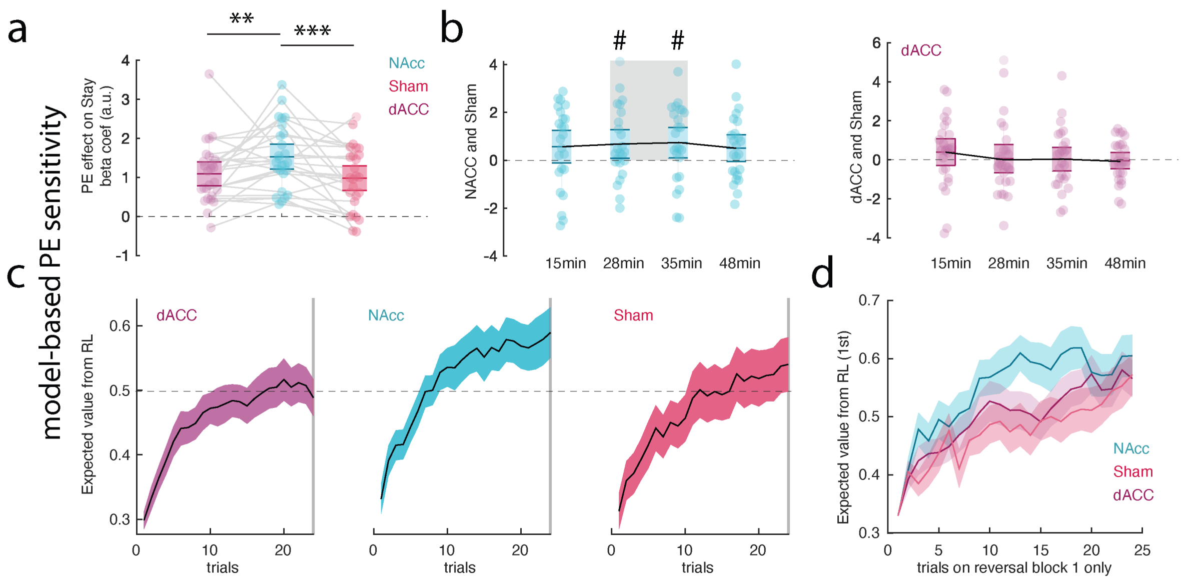

Suppl. Fig. 2. Reinforcement learning model results. a PE–stay analyses revealed an increase in the relationship between PE and subsequent stay behaviours after TUS-NAcc. Each dot represents an individual participant's mean beta estimate for the specified condition (n=26). **b** Left panel: Time course of TUS-NAcc effects on PE-related behaviour, showing the difference between TUS-NAcc and Sham conditions for each of the four post-TUS testing blocks. The TUS-NAcc-induced reward-related changes were most prominent in the middle of the approximately one-hour post-TUS period. This effect was not observed in the right panel, which shows the comparison between TUS-dACC and Sham; no significant time window emerged in that contrast (n=26). **c** Expected value from the RL models curves for the high-probability option across all reversal blocks. A 5-trial running average was applied to smooth trial-by-trial variability, which results in a slight shift in the apparent reversal point—appearing earlier than the actual reversal at trial 24 (trial 25 marks the start of the new reversal period). The shaded area represents the standard error of the mean. **d** Same as **c** but showing only the first reversal block. The three conditions (TUS-NAcc, TUS-dACC, and Sham) are presented stacked and with transparency to allow for direct visual comparison. Exact p values are presented in the supplementary table 5. **a**: statistical significance was determined using One-way ANOVA and two-sided t tests. **b**: single two-sided t-tests were employed for each window. No multiple comparisons were applied.

Supplementary Figure 3

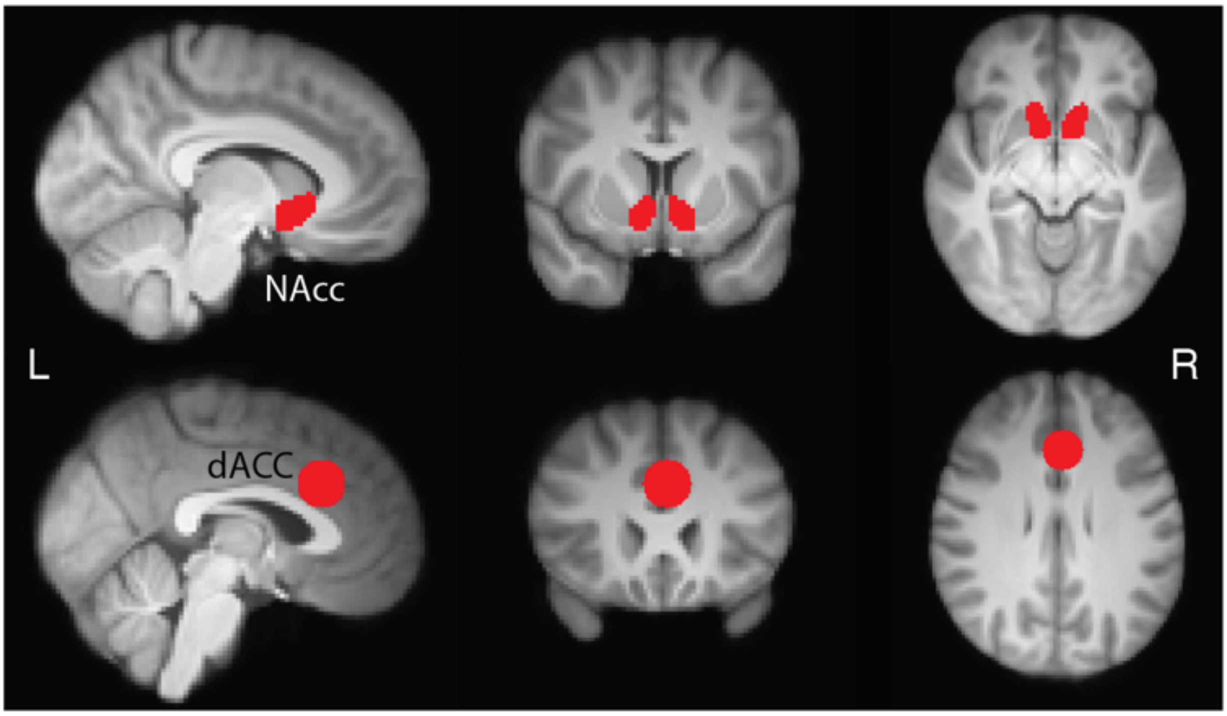

Suppl. Fig. 3. Region of interest (ROI) definition for the ROI analysis reported in the FMRI findings section for both the NAcc and the dACC regions. ROI are presented in red.

Supplementary Figure 4

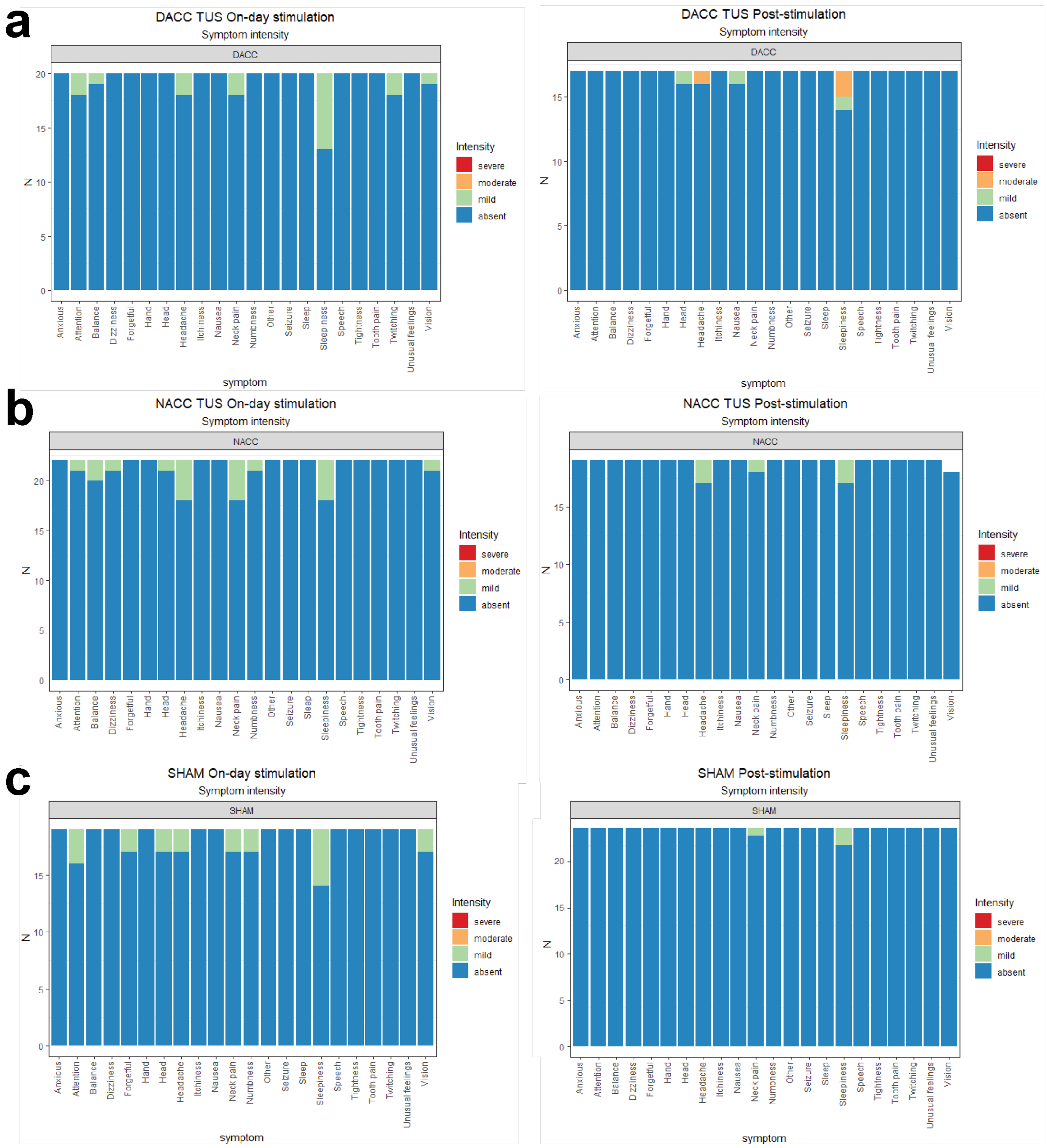

Suppl. Fig. 4. Group safety report. a Total number of responses for all participants (n = 26) from the dACC condition on the day (left) and on the following day (right), coded by the severity of the symptom. **b** Total number of responses for all participants (n = 26) from the NAcc condition on the day (left) and on the following day (right), coded by the severity of the symptom. **c** Total number of responses for all participants (n = 26) from the Sham condition on the day (left) and on the following day (right), coded by the severity of the symptom. Note: Unusual feelings refer to the question asking about experiencing any unusual feelings, attitudes or emotions. This is largely inspired by the work of [6].

Supplementary Table 1 (NAcc)

Suppl. Table 1. Acoustic simulation parameters and output for all study participants in the Nacc condition. The table reports the pressure values at the spatial-peak (Max Pressure), corresponding transcranial mechanical index (MItc) and spatial-peak pulse-average intensity (ISPPA), along with the maximum temperature rise in the head, to assess safety. To evaluate stimulation efficacy, the in situ estimate for the pressure amplitude at the target, Isppa at the target and the size of the -6dB focal volume are reported. Pressure values are given in megapascals (MPa)

Focus depth	Max Pressure (MPa)	MItc	Isppa (W/cm2)	Max. temp. rise	Pressure at target (MPa)	Isppa at target (W/cm2)	-6dB focal volume (mm3)
82	0.44	0.63	6.54	3.37	0.32	3.43	561.13
71	0.64	0.90	13.46	2.14	0.43	6.06	257.75
74	0.66	0.94	14.62	2.31	0.30	2.98	234.75
74	0.66	0.93	14.41	2.70	0.46	6.99	283.63
82	0.62	0.88	12.79	2.39	0.56	10.56	458.13
82	0.61	0.87	12.53	4.59	0.48	7.61	327.00
75	0.62	0.87	12.76	3.07	0.51	8.71	281.75
77	0.61	0.86	12.25	3.61	0.55	10.18	332.00
80	0.63	0.89	13.17	3.05	0.46	7.04	303.63
74	0.65	0.92	14.21	2.83	0.19	1.23	216.00
76	0.64	0.90	13.64	3.10	0.55	10.00	255.50
77	0.52	0.74	9.11	3.13	0.40	5.31	490.25
69	0.66	0.94	14.61	1.34	0.53	9.42	178.50
74	0.53	0.75	9.25	2.46	0.35	4.20	406.25
74	0.59	0.84	11.76	2.11	0.29	2.79	367.63
73	0.63	0.90	13.42	2.59	0.54	9.89	282.63
72	0.60	0.84	11.82	2.19	0.50	8.29	329.00
74	0.59	0.83	11.51	1.35	0.45	6.81	379.38
80	0.56	0.80	10.56	1.89	0.25	2.04	450.38
82	0.63	0.89	13.07	3.88	0.56	10.64	420.38
74	0.64	0.91	13.86	1.71	0.37	4.52	200.38
79	0.67	0.94	14.82	3.27	0.63	13.41	351.75
76	0.57	0.80	10.70	3.04	0.52	9.18	393.25
73	0.64	0.91	13.81	1.71	0.32	3.46	211.00
79	0.61	0.86	12.44	2.50	0.41	5.53	358.63
75	0.68	0.95	15.19	1.82	0.53	9.19	197.25

Supplementary Table 2 (dACC)

Suppl. Table 2. Acoustic simulation parameters and output for all study participants in the dACC condition. The table reports the pressure values at the spatial-peak (Max Pressure), corresponding transcranial mechanical index (MItc) and spatial-peak pulse-average intensity (ISPPA), along with the maximum temperature rise in the head, to assess safety. To evaluate stimulation efficacy, the in situ estimate for the pressure amplitude at the target, Isppa at the target and the size of the -6dB focal volume are reported. Pressure values are given in megapascals (MPa)

Focus depth	Max Pressure (MPa)	MItc	Isppa (W/cm2)	Max. temp. rise	Pressure at target (MPa)	Isppa at target (W/cm2)	-6dB focal volume (mm3)
64.00	0.64	0.90	13.59	1.95	0.59	11.60	175.75
57.00	0.64	0.90	13.61	0.93	0.52	9.01	113.63
57.00	0.52	0.73	8.86	0.94	0.41	5.60	210.38
61.00	0.61	0.86	12.31	1.09	0.49	8.00	175.13
57.00	0.65	0.92	14.06	0.85	0.52	9.01	132.38
58.00	0.57	0.81	10.99	1.01	0.39	5.07	166.13
57.00	0.57	0.80	10.78	0.90	0.37	4.56	167.63
54.00	0.61	0.86	12.28	0.73	0.60	12.00	115.13
58.00	0.47	0.66	7.21	1.21	0.43	6.16	278.38
57.00	0.51	0.72	8.67	1.01	0.37	4.56	201.88
61.00	0.55	0.78	10.26	1.29	0.42	5.88	207.50
58.00	0.53	0.75	9.41	0.88	0.41	5.60	207.00
57.00	0.62	0.87	12.74	0.81	0.53	9.36	131.88
61.00	0.65	0.92	14.03	1.28	0.52	9.01	144.00
61.00	0.64	0.90	13.47	1.39	0.43	6.16	158.75
60.00	0.62	0.88	12.98	1.16	0.51	8.67	165.13
53.00	0.55	0.78	10.09	0.95	0.43	6.16	163.63
59.00	0.61	0.86	12.42	1.00	0.40	5.33	157.25
62.00	0.45	0.63	6.67	1.16	0.35	4.08	92.63
62.00	0.64	0.90	13.54	1.35	0.47	7.36	159.38
65.00	0.49	0.69	7.97	1.63	0.39	5.07	128.25
69.00	0.61	0.87	12.50	1.46	0.48	7.68	84.25
63.00	0.56	0.80	10.58	1.20	0.54	9.72	196.13
60.00	0.65	0.93	14.29	1.09	0.56	10.45	141.63
58.00	0.54	0.76	9.57	1.21	0.38	4.81	196.13
64.00	0.52	0.73	8.98	1.57	0.48	7.68	262.75

Supplementary Table 3

Suppl. Table 3. ANOVA results for no reinforcement learning model: effect of reward on Win–Stay. Post-hoc t-tests revealed a stronger relationship between reward and subsequent win–stay behaviour after TUS-NAcc compared to Sham. No differences were observed for TUS-dACC compared to Sham. The results for each task block are also shown.

ANOVA

Sum of Squares	DF	Mean Squares	F	p-value
6.4468	2	3.2234	3.2954	**0.0424**
73.3613	75	0.9781		
79.8082	77			

Post hoc t-tests

	p-value	CI low	CI high	t-stat	DF	SD
NAcc-Sham	**0.0110**	-0.9550	-0.1364	-2.7461	25	1.0133
NAcc-dACC	**0.0016**	-1.0435	-0.2730	-3.5193	25	0.9537
dACC-Sham	0.5732	-0.2935	0.5186	0.5707	25	1.0054

Difference NAcc and Sham

	p-value	CI low	CI high	t-stat	DF	SD
15min	0.0867	-0.0936	1.2942	1.7894	23	1.6434
28min	**0.0056**	0.3496	1.8249	3.0490	23	1.7469
35min	0.0537	-0.0119	1.3766	2.0330	23	1.6442
48min	0.1133	-0.1453	1.2784	1.6462	23	1.6859

Difference dACC and Sham

	p-value	CI low	CI high	t-stats	DF	SD
15min	0.4118	-0.4827	1.1404	0.8345	25	2.0093
28min	0.6233	-0.5226	0.8553	0.4972	25	1.7058
35min	0.7145	-0.6919	0.9949	0.3699	25	2.0881
48min	0.4832	-0.7650	0.3720	-0.7117	25	1.40764

Supplementary Table 4

Suppl. Table 4. Comparison of learning curves across trials and reversal periods. Participants were more likely to select the high probability option at the end of a reversal period after TUS-NAcc compared to TUS-dACC and Sham. This is particularly true for the first reversal of the block.

All reversal periods

Fixed Effect	Estimate	SE	t-value	DF	p-value
trial	0.0199	0.0026	7.6579	5924	2.19E-14
cond	-0.0113	0.0041	-2.7408	5924	**0.0061**
trial^2	-0.0006	0.0001	-5.0510	5924	4.53E-07

First reversal period only

Fixed Effect	Estimate	SE	t-value	DF	p-value
trial	0.0226	0.0033	6.8422	1478	1.14E-11
cond	-0.0208	0.0052	-3.9714	1478	**7.49E-05**
trial^2	-0.0008	0.0001	-5.3668	1478	9.29E-08

Supplementary Table 5

Suppl. Table 5. ANOVA results for reinforcement learning model: effect of prediction error (PE) on Win–Stay. Post-hoc t-tests revealed a stronger relationship between PE and subsequent win–stay behaviour after TUS-NAcc compared to Sham. No differences were observed for TUS-dACC compared to Sham. The results for each task block are also shown.

ANOVA

Sum of Squares	DF	Mean Squares	F	p-value
4.4277	2	2.2138	3.3304	**0.0411**
49.8554	75	0.6647		
54.2831	77			

Post hoc t-tests

	p-value	CI low	CI high	t-stat	DF	SD
NAcc-Sham	**0.0274**	-0.8249	-0.0532	-2.3433	25	0.9554
NAcc-dACC	**0.0031**	-0.8996	-0.2054	-3.2786	25	0.8593
dACC-Sham	0.5138	-0.2393	0.4662	0.6624	25	0.8733

Difference NAcc and Sham

	p-value	CI low	CI high	t-stat	DF	SD
15min	0.1142	-0.1476	1.2837	1.6421	23	1.6948
28min	**0.0323**	0.0680	1.4064	2.2788	23	1.5849
35min	**0.0360**	0.0482	1.3102	2.2267	23	1.4943
48min	0.0865	-0.0792	1.0995	1.7908	23	1.3957

Difference dACC and Sham

	p-value	CI low	CI high	t-stat	DF	SD
15min	0.2647	-0.3215	1.1201	1.1408	25	1.7847
28min	0.9063	-0.5977	0.6709	0.1189	25	1.5705
35min	0.8685	-0.7021	0.8262	0.1673	25	1.8919
48min	0.8369	-0.4817	0.3933	-0.2080	25	1.0831

Supplementary Table 6

Suppl. Table 6. Comparison of learning curves (that is the rate of choosing the high probability option over the period of one reversal) between TUS-NAcc, TUS-dACC, and Sham.

All reversal periods

Fixed Effect	Estimate	SE	t-value	DF	p-value
trial	0.0300	0.0014	21.9431	7785	**1E-103**
cond	0.0056	0.0029	1.9352	7785	0.0529
trial^2	-0.0009	0.0001	-18.3870	7785	**6E-74**

First reversal period only

Fixed Effect	Estimate	SE	t-value	DF	p-value
trial	0.0192	0.0021	9.3135	1867	**3E-20**
cond	-0.0117	0.0042	-2.7863	1867	**0.0053**
trial^2	-0.0005	0.0001	-5.7059	1867	**1E-08**

Supplementary Table 7

Suppl. Table 7. ANOVA and post-hoc test results for learning rates linked with positive and negative feedback. The learning rates linked with positive feedback were higher after TUS-NAcc compared to dACC. The learning rates associated with negative feedback were not significantly different across the conditions.

ANOVA

Learning rate (positive feedback)

Sum of Squares	DF	Mean Squares	F	p-value
0.3760	2	0.18802	5.6847	**0.0050**
2.4806	75	0.03307		
2.8567	77			

Learning rate (negative feedback)

Sum of Squares	DF	Mean Squares	F	p-value
0.0246	2	0.0123	0.2758	0.7597
3.3508	75	0.0446		
3.3754	77			

Post-hoc tests for learning rate (positive feedback)

	p-value	CI low	CI high	t-stat	DF	SD
NAcc-Sham	**0.0211**	-0.2182	-0.0194	-2.4605	25.0000	0.2462
NAcc-dACC	**0.0060**	-0.2778	-0.0518	-3.0031	25.0000	0.2798
dACC-Sham	0.3432	-0.0521	0.1441	0.9662	25.0000	0.2429

Supplementary Table 8

Suppl. Table 8. ANOVA results for win–stay strategy and maladaptive choices. We found a main effect of condition on the rate of maladaptive choices after TUS-NAcc.

ANOVA
Win–stay after low probability outcome

Sum of Squares	DF	Mean Squares	F	p-value
8.4220	2	4.2110	3.7525	**0.0279**
84.1643	75	1.1221		
92.5864	77			

Single t-test against 0

	p-value	CI low	CI high	t-stat	DF	SD
dACC	0.3988	-0.2630	0.6390	0.8584	25	1.1166
NAcc	**0.0033**	0.2675	1.1978	3.2442	25	1.1515
Sham	0.7646	-0.4126	0.3069	-0.3026	25	0.89079

Supplementary Table 9

Suppl. Table 9. ROI analysis results for PE during reward delivery and reward expectation. There was an increase in the parametric response to reward expectation in the NAcc in the TUS-NAcc condition compared to the Sham and TUS-dACC conditions.

ANOVA
NAcc reward expectation

Sum of Squares	DF	Mean Squares	F	p-value
2.84814906	2	1.4240	7.1545	**0.0014**
14.9283081	75	0.1990		
17.7764571	77			

dACC reward delivery

Sum of Squares	DF	Mean Squares	F	p-value
1.6866	2	0.8433	3.1237	**0.0497**
20.2483	75	0.2699		
21.9349	77			

Post hoc t-tests NAcc reward expectation

	p-value	CI low	CI high	t-stat	DF	SD
dACC-NAcc	**0.0006**	-0.7057	-0.2193	-3.9165	25	0.6021
NAcc-Sham	**0.0248**	-0.5469	-0.0405	-2.3888	25	0.6269
dACC-Sham	0.1918	-0.4280	0.0903	-1.3416	25	0.6416

Post hoc t-tests dACC reward delivery

	p-value	CI low	CI high	t-stat	DF	SD
dACC-NAcc	**0.0076**	-0.6140	-0.1042	-2.9023	25	0.6309
NAcc-Sham	0.2017	-0.4004	0.0889	-1.3110	25	0.6058
dACC-Sham	0.1514	-0.4864	0.0796	-1.4797	25	0.7008

Supplementary Table 10

Suppl. Table 10. Demographic information about the three DBS patients. DBS: deep brain stimulation; OCD: obsessive–compulsive disorder; MDD: major depressive disorder.

Patient	Sex	Age	Psychiatric comorbidities	Bilateral DBS Settings		
				Voltage (V)	Pulse Width (µs)	Frequency (Hz)
1	F	31	OCD	3.5	90	130
2	F	34	OCD<br>MDD	4.0	80	130
3	F	61	OCD<br>MDD	4.0	60	130
Mean (SD)		42 (13.5)				

Supplementary Material 1

Resource Table

RESOURCE	SOURCE	IDENTIFIER
**Deposited data**		
Structural and functional MRI data ("TUS fMRI NAcc part 1-3")<br>Behavioural data	Siti Yaakub<br>Elsa Fouragnan	https://osf.io/j34qz/<br>https://osf.io/w3mev/<br>https://osf.io/vst9y/
**Software and algorithms**		
MATLAB R2023a	Mathworks	https://www.mathworks.com/
FMRIB Software Library v6.0	FMRIB, Oxford, UK	https://fsl.fmrib.ox.ac.uk/
HD-BET	Github	https://github.com/MIC-DKFZ/HD-BET
k-Wave toolbox v1.4	Github	http://www.k-wave.org
MR-to-pCT toolbox v1	Github	https://github.com/sitiny/mr-to-pct
BRIC TUS Simulation Tools v2	Github	https://github.com/sitiny/BRIC_TUS_Simulation_Tools
Reinforcement learning	Github	https://github.com/efouragnan/RL_models
Presentation Neurobs	Presentation	https://www.neurobs.com/
**Other**		
NeuroFUS CTX-500 with optimized steering range (40–80mm)	BrainBox, SonicConcepts	https://brainbox-neuro.com/products/neurofus<br>http://sonicconcepts.com
Stereotaxic neuronavigation system	Brainsight	https://www.rogue-research.com/tms/brainsight-tms/
Siemens Prima (MR)	BRIC MRI	https://www.plymouth.ac.uk/facilities/brain-research-imaging-centre<br>https://www.siemens-healthineers.com/magnetic-resonance-imaging/3t-mri-scanner/magnetom-prisma

Supplementary Material 2

Safety Questionnaire

Please rate the intensity of any symptoms you have had since your stimulation according to the following criteria:
Absent = Not present
Mild = Present but not bothersome
Moderate = Tolerable—required some intervention/medication, but did not interfere with day-to-day activities
Severe = Intolerable—required contact with a GP or hospital A&E

Use the box to the right of each symptom to provide details, e.g., describe what you felt, how long it lasted, medication you took to relieve the symptoms, and whether you think it might be related to the stimulation.

**Since your stimulation, have you had ... ?**	**Intensity of symptom**	**Please provide details**
a headache	☐ absent ☐ moderate ☐ mild ☐ severe	
neck pain	☐ absent ☐ moderate ☐ mild ☐ severe	
tooth pain	☐ absent ☐ moderate ☐ mild ☐ severe	
unusual feelings on your head or scalp	☐ absent ☐ moderate ☐ mild ☐ severe	
itchiness	☐ absent ☐ moderate ☐ mild ☐ severe	
changes to your hearing	☐ absent ☐ moderate ☐ mild ☐ severe	
speech problems	☐ absent ☐ moderate ☐ mild ☐ severe	
vision problems (e.g., double vision)	☐ absent ☐ moderate ☐ mild ☐ severe	
unusual twitching or muscle movement	☐ absent ☐ moderate ☐ mild ☐ severe	
difficulties in balance	☐ absent ☐ moderate ☐ mild ☐ severe	
changes in the movement of your strongest hand	☐ absent ☐ moderate ☐ mild ☐ severe	
numbness or tingling sensations	☐ absent ☐ moderate ☐ mild ☐ severe	
muscle tightness of the face or arm	☐ absent ☐ moderate ☐ mild ☐ severe	
unusual feelings, attitudes or emotions	☐ absent ☐ moderate ☐ mild ☐ severe	
anxiety, worried thoughts or nervousness	☐ absent ☐ moderate ☐ mild ☐ severe	
increased sleepiness	☐ absent ☐ moderate ☐ mild ☐ severe	
changes to your sleep pattern	☐ absent ☐ moderate ☐ mild ☐ severe	
difficulty paying attention	☐ absent ☐ moderate ☐ mild ☐ severe	
increased forgetfulness	☐ absent ☐ moderate ☐ mild ☐ severe	
nausea or sickness to the stomach	☐ absent ☐ moderate ☐ mild ☐ severe	
dizziness or light-headedness	☐ absent ☐ moderate ☐ mild ☐ severe	
a seizure within the last 24 hours	☐ absent ☐ moderate ☐ mild ☐ severe	
other symptoms: ____________________	☐ absent ☐ moderate ☐ mild ☐ severe	

Do you have anything else to report? ________________________

References

1. Boorman, E. D., Behrens, T. E. & Rushworth, M. F. Counterfactual Choice and Learning in a Neural Network Centered on Human Lateral Frontopolar Cortex. *PLOS Biology* **9**, e1001093 (2011).
2. Boorman, E. D., Rushworth, M. F. & Behrens, T. E. Ventromedial Prefrontal and Anterior Cingulate Cortex Adopt Choice and Default Reference Frames during Sequential Multi-Alternative Choice. *J. Neurosci.* **33**, 2242–2253 (2013).
3. Fouragnan, E. F. *et al.* The macaque anterior cingulate cortex translates counterfactual choice value into actual behavioral change. *Nature Neuroscience* (2019) doi:10.1038/s41593-019-0375-6.
4. Wittmann, M. K. *et al.* Global reward state affects learning and activity in raphe nucleus and anterior insula in monkeys. *Nature communications* **11**, 3771 (2020).
5. Priestley, L. *et al.* Dorsal Raphe Nucleus Controls Motivational State Transitions in Monkeys. 2024.02.13.580224 Preprint at https://doi.org/10.1101/2024.02.13.580224 (2024).
6. Legon, W. A retrospective qualitative report of symptoms and safety from transcranial focused ultrasound for neuromodulation in humans. *Scientific Reports* **10**, 5573 (2020).
