# Supplementary material for: Non-invasive ultrasonic neuromodulation of the human nucleus accumbens impacts reward sensitivity
Source: Nat Commun. 2025 Nov 27;16:10192. doi: 10.1038/s41467-025-65080-9 (PMC12660320; doi:10.1038/s41467-025-65080-9)
Supplement: Supplementary file 2 — Reporting Summary [file 41467_2025_65080_MOESM2_ESM.pdf]

nature portfolio

Corresponding author(s): Prof Elsa Fouragnan

Last updated by author(s): 06/08/2025

Reporting Summary

Nature Portfolio wishes to improve the reproducibility of the work that we publish. This form provides structure for consistency and transparency in reporting. For further information on Nature Portfolio policies, see our Editorial Policies and the Editorial Policy Checklist.

Statistics

For all statistical analyses, confirm that the following items are present in the figure legend, table legend, main text, or Methods section.

n/a	Confirmed	
☐	☒	The exact sample size (*n*) for each experimental group/condition, given as a discrete number and unit of measurement
☐	☒	A statement on whether measurements were taken from distinct samples or whether the same sample was measured repeatedly
☐	☒	The statistical test(s) used AND whether they are one- or two-sided<br>*Only common tests should be described solely by name; describe more complex techniques in the Methods section.*
☐	☒	A description of all covariates tested
☐	☒	A description of any assumptions or corrections, such as tests of normality and adjustment for multiple comparisons
☐	☒	A full description of the statistical parameters including central tendency (e.g. means) or other basic estimates (e.g. regression coefficient) AND variation (e.g. standard deviation) or associated estimates of uncertainty (e.g. confidence intervals)
☐	☒	For null hypothesis testing, the test statistic (e.g. *F*, *t*, *r*) with confidence intervals, effect sizes, degrees of freedom and *P* value noted<br>*Give P values as exact values whenever suitable.*
☒	☐	For Bayesian analysis, information on the choice of priors and Markov chain Monte Carlo settings
☒	☐	For hierarchical and complex designs, identification of the appropriate level for tests and full reporting of outcomes
☐	☒	Estimates of effect sizes (e.g. Cohen's *d*, Pearson's *r*), indicating how they were calculated

Our web collection on statistics for biologists contains articles on many of the points above.

Software and code

Policy information about availability of computer code

Data collection	Transcranial ultrasound stimulation was delivered using the NeuroFUS TPO and CTX-500-4 transducer (Brainbox Ltd., Cardiff, UK). Neuronavigation was performed with the Brainsight software v 2.4.11 (Rogue Research Inc., Montréal, Québec, Canada). MRI scans were acquired on a Siemens MAGNETOM Prisma 3T scanner (VE11E, Siemens Healthineers, Erlangen, Germany) with a 32-channel head coil using sequences from the University of Minnesota Centre for Magnetic Resonance Research (CMRR; multiband fMRI: https://www.cmrr.umn.edu/multiband). Behavioral data were acquired using Presentation Neurobs 24.0 on a PC operating computer (https://www.neurobs.com/).
Data analysis	Acoustic simulations were performed using the k-Wave Toolbox (version 1.3) and kArray (version alpha 0.3) tools implemented in MATLAB (R2020b, MathWorks, Inc.). Code for generating pseudo-CT from T1-weighted MR images and for running acoustic simulations as described in this work are available on GitHub: https://github.com/sitiny/mr-to-pct and https://github.com/sitiny/BRIC_TUS_Simulation_Tools. Reinforcement learning analysis and behavioral analyses were performed in MATLAB (R2020b, MathWorks, Inc.) using the code available on GitHub (Github https://github.com/efouragnan/RL_models). FMRI data were pre-processed and analysed using FEAT (FMRI Expert Analysis Tool) and MELODIC tools from the FMRIB Software Library v6.0 (FSL; www.fmrib.ox.ac.uk/fsl).

For manuscripts utilizing custom algorithms or software that are central to the research but not yet described in published literature, software must be made available to editors and reviewers. We strongly encourage code deposition in a community repository (e.g. GitHub). See the Nature Portfolio guidelines for submitting code & software for further information.

Data

Policy information about availability of data

All manuscripts must include a data availability statement. This statement should provide the following information, where applicable:

- Accession codes, unique identifiers, or web links for publicly available datasets
- A description of any restrictions on data availability
- For clinical datasets or third party data, please ensure that the statement adheres to our policy

Data supporting the findings of this study are available on the Open Science Framework at
https://osf.io/j34qz/
https://osf.io/w3mev/
https://osf.io/vst9y/

Research involving human participants, their data, or biological material

Policy information about studies with human participants or human data. See also policy information about sex, gender (identity/presentation), and sexual orientation and race, ethnicity and racism.

Reporting on sex and gender	Sex and gender information were determined based on participant self-reports. Our study included participants of all genders, and the findings are not specific to one sex or gender. In the study design, we aimed to recruit a balanced number of participants from both sexes (14 females and 12 males included in the study). No sex-based analyses were performed as the measured parameters are not known to be influenced by sex.
Reporting on race, ethnicity, or other socially relevant groupings	The study had no focus on race, ethnicity, or other socially relevant groupings; consequently, we endeavored to collect data as inclusively as possible without gathering specific information on these demographics.
Population characteristics	Twenty-six healthy volunteers (14 female; sex and gender aligned by self-report, with no participants identifying as non-binary) aged between 20 and 65 years (mean = 36.3, s.d. ± 13.3) participated in the study. Participants were screened for contraindications to TUS and MRI, had no current diagnosis of neurological or psychiatric disorders, and were free of psychoactive medications at the time of the study.
Recruitment	Participants were recruited through advertisements posted via the University's email circulation lists, on the lab website (www.elsa-fouragnan.com), on social media (Twitter) and through word-of-mouth. Participants received monetary compensation for their participation in the study.
Ethics oversight	The study was approved by the University of Plymouth Faculty of Health Staff Research Ethics and Integrity Committee (reference ID: 2487; date: 13/12/2021).

Note that full information on the approval of the study protocol must also be provided in the manuscript.

Field-specific reporting

Please select the one below that is the best fit for your research. If you are not sure, read the appropriate sections before making your selection.

[x] Life sciences [ ] Behavioural & social sciences [ ] Ecological, evolutionary & environmental sciences

For a reference copy of the document with all sections, see nature.com/documents/nr-reporting-summary-flat.pdf

Life sciences study design

All studies must disclose on these points even when the disclosure is negative.

Sample size	Since there are no previous studies on the behavioral and neural effects of TUS targeted to a deep cortical region, we based our sample size estimate on our previous TUS and MRS study: Yaakub et al., 2023, Nature Communications. We chose the most conservative effect size of 0.8 for our power calculation. We used G*Power (https://www.psychologie.hhu.de/arbeitsgruppen/allgemeine-psychologie-und-arbeitspsychologie/gpower) to conduct a power analysis to determine the sample size. Our goal was to obtain 0.95 power to detect an effect size of 0.8 at the standard 0.05 alpha error probability level. Our power analysis revealed that for a minimum effect size of 0.8, a sample size of 19 is required. These estimates broadly accord with our original predictions but illustrate that we may be close to the limits of detection. In view of these calculations, we have increased the target sample size to 26 participants.
Data exclusions	Exclusion criteria were established before any analyses were performed. fMRI data were excluded if motion exceeded 0.25 mm (relative displacement between consecutive volumes) for more than 50% of the volumes, or if image artifacts were discovered during visual data quality inspection or people had more than 30% missed trials. No data were excluded on this basis.
Replication	All the effects that are reported are ones that were replicated across the group of individuals tested; they were found across the whole group of individuals in a mixed effects analysis. The fMRI results were replicated with and without additional covariates (simulated in situ ISPPA, and simulated TUS focal volume) and the results remained.
Randomization	We used block randomisation to assign each participant to one of six "blocks" that determine the order of the TUS sessions and sham: 1)

sham, dACC, NAcc 2) sham, NAcc dACC, 3) dACC, sham, NAcc 4) dACC, NAcc sham, 5) NAcc sham, dACC, or 6) NAcc dACC, sham. The random list used to create these six blocks was created using the List Randomizer at http://random.org.

Blinding	Participants were blinded but investigators were not blinded.

Reporting for specific materials, systems and methods

We require information from authors about some types of materials, experimental systems and methods used in many studies. Here, indicate whether each material, system or method listed is relevant to your study. If you are not sure if a list item applies to your research, read the appropriate section before selecting a response.

Materials & experimental systems

n/a	Involved in the study
☒	☐ Antibodies
☒	☐ Eukaryotic cell lines
☒	☐ Palaeontology and archaeology
☒	☐ Animals and other organisms
☒	☐ Clinical data
☒	☐ Dual use research of concern
☒	☐ Plants

Methods

n/a	Involved in the study
☒	☐ ChIP-seq
☒	☐ Flow cytometry
☐	☒ MRI-based neuroimaging

Plants

Seed stocks	*Report on the source of all seed stocks or other plant material used. If applicable, state the seed stock centre and catalogue number. If plant specimens were collected from the field, describe the collection location, date and sampling procedures.*
Novel plant genotypes	*Describe the methods by which all novel plant genotypes were produced. This includes those generated by transgenic approaches, gene editing, chemical/radiation-based mutagenesis and hybridization. For transgenic lines, describe the transformation method, the number of independent lines analyzed and the generation upon which experiments were performed. For gene-edited lines, describe the editor used, the endogenous sequence targeted for editing, the targeting guide RNA sequence (if applicable) and how the editor was applied.*
Authentication	*Describe any authentication procedures for each seed stock used or novel genotype generated. Describe any experiments used to assess the effect of a mutation and, where applicable, how potential secondary effects (e.g. second site T-DNA insertions, mosiacism, off-target gene editing) were examined.*

Magnetic resonance imaging

Experimental design

Design type

Evoked fMRI BOLD analysis

Design specifications

Each participant underwent two functional MRI scans during the entire scanning session.

Behavioral performance measures

Participants performed a probabilistic reversal learning task during four blocks of MR acquisitions (two of which were fMRI acquisitions). The task consisted of two runs of 100 trials each (presented during the fMRI scans) and two runs of 60 trials each (no fMRI), giving a total of 320 trials across four blocks. To create trial-wise estimates of expected value (reward prediction) and prediction error, we first used an asymmetric learning model which discriminates based on outcome valence, with two learning rates, one for positive and one for negative feedback. The first analysis in the series was concerned with the relationship between reward and subsequent win–stay behaviours. We thus ran a regression model given by the equation:

WinStay (t+1) ~ Reward (t) + choiceStickniness (t) + isHPScreen (t+1)

where WinStay (t+1) is coded as +1 for Win–Stay, -1 for Win–Switch and 0 otherwise on trial t+1; Reward is coded as 1 if a reward is received and 0 if not on trial t; choiceStickniness is coded as 1 if choice has been repeated and 0 otherwise on trial t; and isHPscreen is coded as 1 if there was a High Probability Symbol on the screen and 0 otherwise. In essence, this is equivalent to looking at the proportion of win–stay behaviours while controlling for other features of the task and behaviour such as any general tendency to choose repetition regardless of reward (choiceStickiness). We then subjected the regression weights to an analysis of variance (ANOVA) such that:

regressionCoef ~ 1 + condition

where condition is a categorical variable (TUS-dACC, TUS-NAcc, or Sham). Further three two-tailed two-sample t-tests were used for all possible TUS-dACC, TUS-NAcc and Sham pairs applying Bonferroni correction.

The second analysis tested the difference between the regression weights from Analysis A for NAcc vs. Sham and dACC vs. Sham for each of the four blocks (2 fMRI and 2 tasks only). As these blocks were acquired at different times, this allowed us to plot the effect identified in Analysis A over time. We used two-tailed paired t-tests to check significance of this difference for each block and considered $p < 0.05$ to be significant. We did not perform multiple comparisons as we used predefined hypotheses and tests.

The third analysis looked at the rate of choice towards the high probability symbol, for the window significant in Analysis

B, for the runs within a block, averaged across blocks, and then during the first run of a block, averaged across blocks. We then repeated the Analyses A, B, and C with model estimates from the RL model. In Analysis A, we used the prediction error at t instead of the reward at t. In Analysis C, we used the expected value associated with the high probability symbol instead of the choice rate associated with the high probability symbol and presented it for all four blocks.

Acquisition

Imaging type(s): Structural and functional

Field strength: 3 Tesla

Sequence & imaging parameters: MRI scans were acquired on a Siemens MAGNETOM Prisma 3T scanner (VE11E, Siemens Healthineers, Erlangen, Germany) with a 32-channel head coil. The scans in this study included a T1-weighted MPRAGE sequence acquired in the sagittal plane (repetition time (TR) = 2100 ms, echo time (TE) = 2.26 ms, inversion time = 900 ms, flip angle (FA) = 8°, GRAPPA acceleration factor = 2, field of view = 256 × 256 mm, number of slices = 176, voxel size = 1 × 1 × 1 mm3), two GE-EPI fMRI scans during which participants performed the probabilistic reversal learning task lasting approximately 10-minutes each (acquisition plane tilted 30° clockwise from the line parallel to the AC-PC line, 1400 ms TR, 30 ms TE, 67° FA, 2.5 mm slice thickness, no slice gap, multi-band acceleration factor of 2, and 60 interleaved slices of 96 × 96 matrix size, giving a voxel size of 2.5 × 2.5 × 2.5 mm3), and field maps for fMRI distortion correction.

Area of acquisition: A whole brain scan was used for structural and functional MRI acquisitions.

Diffusion MRI: ☐ Used ☒ Not used

Preprocessing

Preprocessing software: fMRI preprocessing was performed using tools from the FMRIB Software Library v6.0 (FSL; www.fmrib.ox.ac.uk/fsl). Pre-processing included MCFLIRT motion correction, B0 inhomogeneity correction (Effective EPI echo spacing = 0.49 ms, EPI TE = 30 ms, unwarp dif = -y, signal loss thr = 10%), brain extraction, spatial smoothing (5 mm FWHM), highpass filtering (0.01 Hz).

Normalization: fMRI data were normalised to the MNI standard space via a linear transform (FSL FLIRT) to each individual's high-resolution T1-weighted MRI and a non-linear transform to the MNI152 template (FSL FNIRT).

Normalization template: MNI152_T1_2mm_brain (as packaged with the FSL software)

Noise and artifact removal: Motion outliers were identified using the fslmotionoutliers tool and were included as nuisance covariates in the model.

Volume censoring: We did not remove volumes during which significant movement occurred, instead, we used our motion-related artifacts (i.e. regression of motion parameters) as regressors of non interest that were not convolved in our general linear models.

Statistical modeling & inference

Model type and settings: We employed a mass univariate approach within the general linear model framework to perform whole-brain statistical analyses as well as region of interest analyses of functional data as implemented in the FMRIB Software Library. We ran a single-group tripled t-test which corresponds to a repeated measures ANOVA with one fixed factor with three levels and one random factor. Fitting such a mixed effects model with ordinary least squares (OLS) (as implemented in FEAT) requires an assumption of compound symmetry. This is the state of equal variance and intra-subject correlations being equal. That is, Cov (scan1,scan2) = Cov(scan1,scan3) = Cov(scan2,scan3). For these whole-brain fMRI results, all images were thresholded given a one-sided t-test and subsequently FDR-corrected at $p < 0.05$.

Effect(s) tested: The effect tested was a measure of whole-brain functional connectivity of the seed region of interest derived from the voxel-wise whole-brain general linear model described above.

Specify type of analysis: ☐ Whole brain ☐ ROI-based ☒ Both

Anatomical location(s): We hypothesised that we would find reward-related BOLD changes within the targeted areas of TUS neuromodulation. For the NAcc site, we used the bilateral NAcc defined anatomically by the probabilistic Harvard–Oxford subcortical structural atlases. For the dACC site, we created a sphere around the maximum peak TUS intensity (ISPPA in situ) across participants extracted from our acoustic simulation. This is presented in the supplementary material).

Statistic type for inference (See Eklund et al. 2016): Whole-brain Z-statistic maps were thresholded using clusters determined by a one-sided t-test (corresponding to a $p = 0.05$) and a FDR cluster significance threshold of $p = 0.05$.

Correction: FDR-corrected cluster significance threshold of $p = 0.05$

Models & analysis

n/a	Involved in the study
☐	☒ Functional and/or effective connectivity
☒	☐ Graph analysis
☒	☐ Multivariate modeling or predictive analysis

Functional and/or effective connectivity	The parameter estimate derived from the voxel-wise whole-brain general linear model was used as a measure of seed-based functional connectivity.
